# Discovery of an aquaporin (CrAQP2) in the freshwater larval midge, *Chironomus riparius* and its role in response to road de-icers

**DOI:** 10.1016/j.cris.2026.100123

**Published:** 2026-01-16

**Authors:** Britney N. Picinic, Amber Reinsborough, Sima Jonusaite, Jean-Paul V. Paluzzi, Andrew Donini

**Affiliations:** a Department of Biology at York University, Toronto, ON M3J 1P3, Canada; b Department of Biological Science at The University of Tulsa, Tulsa, OK 74104, USA

**Keywords:** Osmoregulation, Malpighian tubules, Anal papillae, Brine-beet juice de-icer, Salinization

## Abstract

*Chironomus riparius* midge larvae are ubiquitous in freshwater ecosystems throughout the Northern Hemisphere. Recently, freshwater in temperate regions has experienced a threat from salinization due to the use of road de-icers. As ambient temperatures fluctuate, snow and ice melt carry the de-icers into nearby freshwater and significantly raise salt levels. Recently, organic de-icers including brine beet juice de-icer (BBJD) have been implemented as an “ecofriendly” alternative to traditional road salt. Not much is known about the effects of BBJD on freshwater invertebrates. Aquatic insects respond to salinity by adjusting the ion transport functions of osmoregulatory organs. A key component in insect osmoregulation is the presence of water channel proteins known as aquaporins (AQPs) that allow movement of water along an osmotic gradient. To date, there is limited knowledge on the effects of salinity on AQP function in aquatic insects. In this study, we characterized a water-specific AQP known as CrAQP2 (a PRIP homolog) in the osmoregulatory organs of *C. riparius* larvae. CrAQP2 was immunolocalized in osmoregulatory organs with greatest transcript abundance in the Malpighian tubules. NaCl caused differential *Craqp2* transcript expression in some of the organs, whereas BBJD had little effect on *Craqp2* transcript levels. *Craqp2* knockdown decreased total body water regardless of treatment and reduced survival of larvae in BBJD and NaCl. Therefore, CrAQP2 appears to be important in maintaining total body water levels stable and likely plays a role in the ability of midge larvae to respond to salinity.

## Highlights

• CrAQP2 is expressed in anal papillae and throughout the alimentary canal of the midge.
• CrAQP2 expression is altered in response to NaCl in various osmoregulatory organs.
• Knockdown of CrAQP2 reduces survival of midges in de-icer contaminated water.
• Beet juice de-icer causes similar effects on *C. riparius*, relative to NaCl in water.

## 1 Introduction

The study of freshwater fauna has been a popular topic due to the unique diversity seen in freshwater ecosystems. Inland waters harbour a wide variety of animals, with over 60 % of those animals being aquatic insects (Dijkstra et al., 2014). The ecological role of aquatic insects is important as they act as primary consumers, detritivores, and predators (Dijkstra et al., 2014). In particular, the presence of aquatic detritivorous insects is essential to freshwater ecosystems because they feed on decaying matter and play a critical role in decomposition and nutrient cycling within their ecosystems. One of many detritivores and primary consumers is the dipteran Chironomidae species, *Chironomus riparius*, which is a non-biting freshwater midge that is widely distributed throughout the Northern Hemisphere (Pinder, 1986; Nemec et al., 2012).

Larval *C. riparius* reside in freshwater aquatic environments as benthic organisms and their post-embryonic development involves four larval instar phases before pupating and emerging as terrestrial adults. The larvae have been effectively used in toxicology studies for freshwater ecosystems (Vogt et al., 2011; Nicacio and Juen, 2015; Manfrin et al., 2018). *C. riparius* larvae have been shown to tolerate moderate increases in salinity (Jonusaite et al., 2010). This is significant because in recent decades, anthropogenic activities including but not limited to, the application of fertilizers, resource mining, and the use of road de-icers have resulted in increased levels of salts in freshwater ecosystems. These activities have thus been identified as deadly threats to freshwater ecosystems (Shuler and Relyea, 2018). In the Northern Hemisphere where *C. riparius* is widely distributed, the use of road de-icing agents through the winter months is important in preventing ice build-up on roadways but leads to freshwater salinization. Typically, the de-icers used are in the form of NaCl pellets or NaCl brine. However, as temperatures fluctuate in these northern regions, the snow and ice melt carries the NaCl with it into the nearby freshwater ecosystems. It has been shown that road salt runoff and increased salinity can have detrimental effects on freshwater organisms that inhabit these aquatic ecosystems due to elevated levels of Na^+^ and Cl^-^ (Meter et al., 2011; Nowghani et al., 2019; Hintz et al., 2022; Dugan and Arnott, 2022). As a potentially eco-friendly alternative to road salt, brine-beet juice de-icers (BBJD) containing relatively lower levels of NaCl are being implemented in limited amounts as replacement to the traditional NaCl de-icers (Nutile and Solan, 2019; Picinic et al., 2022; Reinsborough and Donini, 2025). However, as the BBJD is derived from sugar beets, it is naturally high in K^+^-levels, with reportedly at least 7-fold greater levels of K^+^ in comparison to what is found in NaCl-based de-icers (Fay and Shi, 2012; Wruss et al., 2015; Picinic et al., 2022; Reinsborough and Donini, 2025). The impact of K^+^ on freshwater animals warrants further investigation before widespread use of BBJD as a de-icing agent since it has been documented that half the concentration of KCl compared to NaCl is lethal to at least one chironomid species (Hamilton et al., 1975). In previous studies, it has been shown that increasing concentrations of BBJD in the surrounding media of a freshwater bivalve causes body K^+^ levels to drastically rise (Gillis et al., 2021). More recently, it was shown that BBJD may be equally harmful compared to the traditional NaCl-based de-icer to the amphipod crustacean, *Hyalella azteca*, due to increasing levels of ions in the water which impacted the activity of important enzymatic ion-transporters (Picinic et al., 2022). Furthermore, BBJD caused significant alterations in the osmoregulatory physiology of *C. riparius*, which reduced K^+^ uptake and favoured K^+^ secretion (Reinsborough and Donini, 2025).

Aquatic insects regulate salt and water balance through the function of several specialized osmoregulatory organs found in the alimentary canal that work collectively. The gastric caeca (GC) are implicated in ion and water absorption into the haemolymph (Dow, 1987; Clark et al., 1999; Jonusaite et al., 2013; Linser and Dinglasan, 2014; D’Silva et al., 2017). The midgut (MG) is primarily involved in food digestion, nutrient absorption, but also plays a role in ion and water absorption (Dow, 1987; Clark et al., 1999; Jonusaite et al., 2013; Linser and Dinglasan, 2014). The Malpighian tubules (MTs) function in conjunction with the hindgut (HG) to produce urine that favours water secretion and ion conservation in freshwater aquatic insects, driven by the active transport of ions and movement of water via osmosis (Clark and Bradley, 1998; Donini et al., 2006; Zadeh-Tahmasebi et al., 2016). To achieve this, the ion rich primary urine secreted by the MTs is passed to the HG where final alterations of the urine such as the reabsorption of ions can occur, especially in a freshwater environment (Bradley and Phillips, 1977; Jonusaite et al., 2013; Linser and Dinglasan, 2014). Some larval-stage insects including *C. riparius* and the mosquito *Aedes aegypti*, possess externally localized osmoregulatory organs known as the anal papillae (AP). The AP are balloon-shaped organs comprised of a single-cell layer thick epithelium that is primarily involved in active ion uptake from the environment in freshwater aquatic insects (Jarial, 1995; Nguyen and Donini, 2010; Surendran et al., 2018). In addition, many aquatic insects also possess ionocytes whether on their gills or their integument, which function similarly to the AP for ion uptake in freshwater environments (Cochran et al., 2024). All together, these osmoregulatory organs work to regulate the ionic composition and osmolarity of body fluids and play an important role in the ability of aquatic insects to tolerate changes in environmental salinity.

To date, the effects of salinization on ion transport in freshwater insects has been studied and reviewed (Silver and Donini, 2021); however, comparatively much less is known about how salinization affects the transport of water. A key component in regulating and maintaining water levels in freshwater insects is the presence of water channel proteins, known as aquaporins (AQPs). AQPs are characterized as transmembrane domain proteins that form a channel in which water can traverse cellular membranes according to the osmotic gradient (Campbell et al., 2008; Spring et al., 2009; Drake et al., 2015; Picinic et al., 2024). AQPs have been characterized in other dipteran insects such as the fruit fly *Drosophila melanogaster* (Dow and Davies, 2001; Kaufmann et al., 2005; Campbell et al., 2008) and the mosquito *Ae. aegypti* (Duchesne et al., 2003; Drake et al., 2010, 2015; Akhter et al., 2017; Misyura et al., 2017, 2020; Picinic et al., 2024). Additionally, some AQP genes have been identified in midges such as the sleeping chironomid *Polypedilum vanderplanki* (Kikawada et al., 2008) and the non-flying Antarctic midge *Belgica antarctica* (Yi et al., 2011); however, there is currently no information about AQPs in the freshwater midge, *C. riparius*.

Of the six known insect AQP genes, AQP1 and AQP2, which are named following nomenclature established previously (Drake et al., 2010), have been identified as water-specific AQPs that have a high selectivity for water transport (Drake et al., 2015). AaAQP1 has been localized in larval *Ae. aegypti* to key osmoregulatory organs including the GC, MG, MTs, HG, and AP (Marusalin et al., 2012; Misyura et al., 2020). The AQP1 homolog in *B. antartica* was also found to be localized to the FG, MG, and MTs (Yi et al., 2011), alluding to its important function in water transport in these various osmoregulatory organs. Additionally, AaAQP2 has also been localized to the GC, MG, MT, HG, and AP of larval *Ae. aegypti* (Marusalin et al., 2012; Misyura et al., 2020) and its homolog has been immunolocalized in the FG, MG, and HG of the midge *B. antarctica* (Yi et al., 2011). Furthermore, AaAQP2 has been shown to be salinity responsive in the AP of larval *Ae. aegypti*, where increased salinity in brackish water conditions caused a significant increase in AaAQP2 transcript and protein levels relative to freshwater conditions (Durant et al., 2021).

In order to begin to understand how both NaCl and BBJD may affect the regulation of water transport in freshwater insects we identified an important putative water channel protein CrAQP2, a homolog of the insect AQP2, and studied its expression in the osmoregulatory organs of *C. riparius* exposed to NaCl or BBJD. We hypothesized that CrAQP2 transcript and protein would be abundant in each of the osmoregulatory organs of *C. riparius*, with highest expression expected in the MTs and AP where ion and water transport are constant in hypoosmotic conditions. Additionally, we predicted that in the presence of the de-icers, *Craqp2* would be upregulated in the organs that typically function in water absorption such as the MG and AP; whereas *Craqp2* was predicted to be downregulated in organs that typically function in water secretion such as the GC and MTs. Finally, we hypothesized that the reduction of *Craqp2* transcript expression in the midge larvae by RNA interference would result in increased mortality when larvae were faced with abrupt transfer to salinity on account that they would have difficulty regulating body water levels.

## 2 Materials and methods

### 2.1 Animal rearing

*Chironomus riparius* third and fourth instar larvae were taken from a laboratory colony that is maintained at York University (Jonusaite et al., 2013). Larvae developed in ∼6 L tanks containing ∼3 cm of sand (K&E Industrial Sand, ON, Canada) and 3–4 L of artificial fresh water (AFW: 300μ M NaCl, 300μ M NaHCO_3_, 380μ M CaCl_2_, 43μ M KCl; pH 7.6). The tanks were aerated with air stones connected to a constant air supply. The larvae were fed every 2–3 days with crushed TetraFin Goldfish Flake Food (Tetra Holding US, VA, USA) and kept at 21 to 23 °C on a 12h:12 h light: dark schedule. The water in the tanks was topped up once a week with AFW and water changes were completed every 3–4 weeks. Any potential accumulation of salts from the dilute AFW was measured to be negligible. For treatment with de-icers, 2 % brine beet juice de-icer (BBJD) and 7 g/L NaCl-contaminated water (SCW) were chosen based on previously established LC_50_ curves, which determined 50 % survival at each of those concentrations for late-stage *C. riparius* larvae (Reinsborough and Donini, 2025; Jonusaite et al., 2013). To the best of our knowledge, there is no method for measuring the amount of BBJD that enters waterways but, in the case of NaCl, chloride levels ranging from ∼ 4 to 7 g/L and as high as 10.3 g/L have been recorded in bodies of water near roads (Corsi et al., 2010; Hintz et al., 2022; Kaushal et al., 2005).

### 2.2 **Identification of Cr**AQP**2 tissue collection, and quantification of Cr**AQP**2 expression with qPCR**

To identify *Craqp2* in *C. riparius* larvae, the *C. riparius* reference genome PGI_CHIRRI_v4 (GCA_917,627,325.4; Schmidt et al., 2020) was searched by tblastn operation using the AQP2 (PRIP) deduced protein sequences of *Aedes aegypti* (AY433444), *Polypedilum vanderplanki* (AB281619), and *Belgica antarctica* (AB602340) through the National Center for Biotechnology Information (NCBI) database. Primers were then designed to target a large portion of the putative *Craqp2* gene (Table 1). To obtain total RNA samples, 3–4 fourth instar larval *C. riparius* were taken from the lab colony and transferred to a chilled 300μl aliquot of lysis buffer, prepared from the PureLink RNA minikit (Invitrogen, MA, USA). RNA extraction on whole body samples was completed following the protocol provided in the PureLink RNA minikit (Invitrogen). RNA samples were purified using the TURBO DNAfree^࣪^ Kit (Applied Biosystems, ON, Canada) and cDNA was then synthesized using the iScript cDNA synthesis kit (BioRad, CA, USA) consisting of both oligo(dT) nucleotides and random hexamer primers. The cDNA template was then amplified with Q5® High-Fidelity DNA Polymerase (New England Biolabs, ON, Canada) using specific *Craqp2* primers (Table 1) and amplicon band size was confirmed on an agarose gel. The amplified product was then purified using the Monarch PCR Purification Kit (New England Biolabs, ON, Canada), concentration was determined using a NanoDrop 2000 spectrophotometer (Thermo Scientific, DE, USA) and then samples were sent for Sanger sequencing (The Centre for Applied Genomics, Sick Kids, ON, Canada). Sequencing quality and base accuracy was analyzed using Geneious Software (Dotmatics, MA, USA). Following this, quantitative real time PCR (qPCR) primers were designed from the confirmed partial gene sequence, targeting exon-exon boundaries to ensure cDNA amplicon specificity (Table 1).

**Table 1:** Oligonucleotides used for *Chironomus riparius* AQP2 gene amplification.

**Oligo Name**	**Forward Primer Sequence**	**Reverse Primer Sequence**	**Expected Band Size**	**Experiment Used For**
**CrAQP1**	CACTGGGTGTTGACGAGATC	TAAAGAGATGGGCGCACTTC	780bp	PCR
***CrAQP2* qPCR**	TGCTATGACTATTGGACATG	ACATGTAGTGCTGCAGTTCC	153bp	qPCR
**40S Reference Gene**	TGTACAGAAGAAGAGGTCGAGAAA	GCATACCACGATGAGCACG	230bp	qPCR
**RP11 Reference Gene**	CCGTACATTGCACAGTTCGT	CATTATAGCCAGGTCTTCCC	216bp	qPCR
**Elf1 Reference Gene**	GCTCCTGGACATCGTGATTT	TGACTCCCAATGTGAAAGCC	159bp	qPCR
**Tubulin Reference Gene**	ACTTCTGCTTCAAAATGCGTG	CTACGGTTGGTTCCAGATCG	237bp	qPCR
**CrAQP2 dsRNA**	CACTGGGTGTTGACGAGATC	GCAACACTTCCAAAACATTGAGC	304bp	dsRNA Synthesis
**dsAQP2 Reverse T7**	CACTGGGTGTTGACGAGATC	TAATACGACTCACTATAGGGGCAACACTTCCAAAACATTGAGC	320bp	dsRNA Synthesis
**dsAQP2 Forward T7**	TAATACGACTCACTATAGGGCACTGGGTGTTGACGAGATC	GCAACACTTCCAAAACATTGAGC	320bp	dsRNA Synthesis
**BLac dsRNA**	ATTTCCGTGTCGCCCTTATTC	CGTTCATCCATAGTTGCCTGAC	800bp	dsRNA Synthesis

Each oligo name is listed with the corresponding forward and reverse primer sequences used to produce each amplicon. Oligonucleotides used for AQP2 gene identification and amplification are shown and were used for PCR, qPCR (RT-PCR), and dsRNA synthesis.

*Craqp2* transcript levels were then measured in the alimentary canal of AFW-reared larval *C. riparius* including the foregut with gastric caeca (FG+GC), midgut (MG), Malpighian tubules (MTs), hindgut (HG), and anal papillae (AP). The organs were isolated and pooled from ∼40 larvae on ice in 200μl of chironomid saline (5 mM KCl, 74 mM NaCl, 1 mM CaCl_2_, 8.5 mM MgCl_2_, 10.2 mM NaHCO_3_, 8.6 mM HEPES, 20 mM glucose, 10 mM glutamine), which were dissected in a Sylgard®-lined petri dish filled with the same saline, constituting one biological sample. Following collection, organ samples were centrifuged at 10,000 *g* for 1 min at RT, the saline was removed, and 1 mL of TRIzol® reagent (Invitrogen, ON, Canada) was added. Samples were sonicated on ice using an XL 2000 Ultrasonic Liquid Processor (QSONICA - LLC, CT, USA) and total RNA was isolated from the lysed organ samples following the manufacturer’s guidelines. The resulting RNA samples were treated with the TURBO DNAfree^࣪^ Kit for removal of any potential genomic DNA (Applied Biosystems, ON, Canada). RNA sample concentrations and purity values were measured using a NanoDrop 2000 spectrophotometer (Thermo Scientific, DE, USA). cDNA for each organ was synthesized using the iScript cDNA synthesis kit (BioRad, CA, USA), as previously stated and following the manufacturer’s instructions. For examining of the effects of de-icers on *Craqp2* transcript abundance in each of the osmoregulatory organs previously listed, midge larvae were taken from the lab colony and placed in 100 mL of either BBJD, SCW or AFW (control) for 24hr acute exposures. Following that, all samples were treated identically for RNA isolation and cDNA synthesis (as described above), and for subsequent qPCR. *Craqp2* transcript abundance in AFW, BBJD, and SCW-treated midge larvae was determined with qPCR conducted using a CFX Duet Real-Time PCR System (Bio-Rad Laboratories, ON, Canada) in combination with the SsoAdvanced Universal SYBR Green Supermix (Bio-Rad Laboratories, ON, Canada), following the manufacturer’s instructions. Relative *Craqp2* transcript abundance was calculated using the previously established ΔΔC_T_ method (Livak and Schmittgen, 2001) and for the expression profile, values were normalized to the geometric mean of *Rp11* and *40S* reference genes. For individual *Craqp2* expression in organs exposed to the de-icers, *Rp11* and *40S* reference genes were used for the GC+FG, MG, HG, and AP, whereas tubulin and *Elf1* were used for the MTs as a result of variations in gene transcript in the MTs with BBJD and SCW (Table 1). All reference genes were confirmed to show no changes in transcript abundance with each treatment including AFW, BBJD, and SCW, in each of the organs studied with the respective sets of reference genes. The expression profile across the osmoregulatory organs in freshwater involved 3–4 biological replicates, while the individual *Craqp2* expression analyses for each organ in response to road de-icers involved 4–6 biological replicates, all loaded as technical triplicates for quantitative accuracy.

### 2.3 Immunohistochemistry

Immunohistochemistry (IHC) was carried out on whole body (WB) *C. riparius* midge larvae to localize CrAQP2 in the alimentary canal. Midges were taken from the lab established colony and placed in 100 mL of AFW, BBJD, or SCW for 24hr acute exposures. Midges were placed in Bouin’s fixative for 2hr at room temperature followed by a replacement of fixative with 70 % ethanol and kept at 4 °C until ready for dehydration. For each treatment, 3–4 biological replicates were used, in addition to ∼4 technical replicates for confirmation of immunostaining consistency. The IHC procedures carried out in this study followed previously established protocols (Akhter et al., 2017; Misyura et al., 2020; Sajadi et al., 2023; Picinic et al., 2024). Briefly, fixed WB samples were embedded in paraffin wax and then ∼5μ m sections were cut using an Epredia HM325 manual microtome (Epredia, MI, USA). Sectioned tissues were then placed on Fisherbrand࣪ ColorFrost࣪ Plus Adhesion Microscope slides, with each slide having tissue sections from the AFW, BBJD, and SCW samples, adjacent to each other. Slides were then processed in a two-day procedure where tissue sections were rehydrated and repeatedly washed with 1xPBS. All slides were probed with a previously described affinity purified polyclonal antibody raised against a peptide representing a small fragment of the *Aedes aegypti* AQP2 homolog (AaAQP2) (1:50 rabbit polyclonal antibody raised against the peptide CNGLGNTGLKENVQD, Genscript, NJ, USA) (Durant et al., 2021; Picinic et al., 2024). An alignment between the custom AaAQP2 antibody antigen sequence and the putative CrAQP2 antigen sequence revealed ∼50 % similarity (see Supplementary Figure S1). The mouse monoclonal anti-a5 antibody for Na^+^/K^+^-ATPase (NKA) (Douglas Fambrough, Developmental Studies Hybridoma Bank, IA, USA, 1:5 dilution) was used as a membrane marker for all tissue sections (Picinic et al., 2024). The secondary antibody used to detect AaAQP2-like protein was a goat anti-rabbit AlexaFluor 594 conjugated (Jackson Immunoresearch) antibody used at a 1:400 dilution and the secondary antibody used to detect NKA was a goat anti-mouse secondary antibody conjugated to Cy2 (Jackson Immunoresearch) used at a 1:500 dilution. To determine antibody specificity, control slides were prepared, where the AaAQP2 primary antibody was omitted from the processing step and only secondary antibody was added. Control and experimental slides were treated identically, where tissue sections from AFW, BBJD, and SCW samples were placed adjacent to each other on each slide and ran through the same IHC processing protocol together. Slides were then mounted with 1:1 of glycerol:1xPBS mounting media containing 4μ*g*/mL DAPI and left to dry for at least 24hr before imaging. Fluorescent microscopy was completed using an Olympus IX81 fluorescent microscope (Olympus Canada, ON, Canada) and images were captured using CellSense® 1.12 Digital Imaging software (Olympus Canada). Identical acquisition settings were utilized for all tissue sections on each individual slide, to ensure comparisons could be made between AFW, BBJD, and SCW tissue samples. Images were overlayed using ImageJ 1.53k software.

### 2.4 **dsRNA synthesis and confirmation of Cr**AQP**2 knockdown**

To obtain whole body RNA samples, 3–4 fourth instar larval midges were transferred to a chilled 300μl aliquot of lysis buffer, prepared from the PureLink RNA minikit (Invitrogen, MA, USA) and RNA extraction, purification, and cDNA synthesis was completed as described above. To obtain a large amount of *Craqp2* cDNA serving as template for dsRNA synthesis*,* PCR reactions with the Q5 Polymerase (New England Biolabs, ON, Canada) using specific *Craqp2* primers designed to target a relatively large portion of the gene (Table 1). The amplified product was then purified using the Monarch PCR Purification Kit (New England Biolabs, ON, Canada) and its concentration was determined using a NanoDrop 2000 spectrophotometer (Thermo Scientific, DE, USA). dsRNA synthesis was completed following a previously established protocol (Chasiotis et al., 2016; Misyura et al., 2017). The T7 promoter sequence was added to the 5′ end of forward and reverse primers used to amplify a portion of the *Craqp2* gene. In addition, as a knockdown control gene, a fragment of the bacterial β-lactamase (*βLac*) gene was amplified from a pGEM-T-Easy vector using previously established primers (Chasiotis et al., 2016) and *βLac* primers with T7 promoter sequence were used (Table 1). Separate reactions were run which included the dsRNA cDNA template with either the appropriate forward primer and the reverse primer with a T7 promoter sequence or the forward primer with T7 promoter sequence and the appropriate reverse primer. Following amplification, matching reactions were pooled and then purified with the Monarch PCR Purification kit (New England Biolabs, ON, Canada). The amplicons containing T7 promoter sequences were then used to make dsRNA for *Craqp2* and *βLac* with the Promega T7 RiboMAX Express RNAi System Kit (Promega, WI, USA), following the manufacturer’s instructions. Delivery of dsRNA for *Craqp2* or *βLac* involved feeding larval midges following a protocol previously established for larval *Ae. aegypti* knockdown (Singh et al., 2013; Chasiotis et al., 2016; Durant and Donini, 2018) with minor modifications. Specifically, for each biological replicate, ∼10–12 third and fourth instar larvae were taken from the lab colony and placed in 150μl of RNAse-free water containing 0.5μ*g*/μl dsRNA (*Craqp2* or *βLac*), left to incubate in an open microcentrifuge tube for 2hr at room temperature, before being transferred to 100 mL of aerated AFW. Larvae were left in the aerated AFW containers for 48hr and 72hr post dsRNA feeding before 4–5 whole body larvae were sonicated in chilled lysis buffer, total RNA was extracted, and cDNA synthesized as described above. To confirm knockdown, qPCR on the cDNA samples was performed as described above and the relative transcript abundance in 48hr post *Craqp2* dsRNA feeding was normalized to the *dsβLac* treatment group.

### 2.5 Body water

Potential effects of *Craqp2* knockdown on total body water of *C. riparius* larvae under osmotic challenges was measured following a previously established protocol (Nguyen and Donini, 2010). Larvae were placed in microcentrifuge tubes at a density of ∼10 larvae/150μl of 0.5μ*g*/μl ds*AQP2* or ds*βLac* for 2hr at room temperature, as described above and then transferred into aerated containers of AFW for 48hr. Larvae were equally distributed into containers containing 100 mL of AFW, BBJD, or SCW for a 24hr acute exposure. Following this exposure, individual midges were dried on a KimTech wipe and placed into individual pre-weighed PCR tubes where their wet weight was measured using a microbalance (model UMXP; Mettler-Toledo International, ON, Canada). Larvae in their individual PCR tubes were placed in an oven set to 60 °C for ∼5 days. Larvae were weighed again in their individual PCR tubes to obtain their dry weight and the difference between wet weight and dry weight was calculated to determine total body water. For AFW *n* = 6–8; BBJD *n* = 7–10; SCW *n* = 7–10.

### 2.6 Midge survival

To assess the survivability of larval *C. riparius* under osmotic challenges post *Craqp2* knockdown, three rounds of survival assays were completed. For each round, 30 larvae were placed in microcentrifuge tubes at a density of ∼10 larvae/150μl of 0.5μ*g*/μl ds*AQP2* or ds*βLac*. The microcentrifuge tubes were kept open at room temperature for 2hr. Midges were transferred to containers with 100 mL of aerated AFW, for ds*AQP2* and ds*βLac* individuals, respectively and held for 48 hrs. Then, the group of larvae were evenly distributed into to 200 mL of AFW as the control, or the treatment groups with either BBJD or SCW, all with constant aeration. The midge larvae were then left in their treatments for 7 days, with daily survival counts completed. For ds*βLac*-treated individuals, the total number of larvae assessed was *n* = 17 for AFW, *n* = 18 for BBJD, and *n* = 17 for SCW. For ds*AQP2*-treated individuals, the total number of larvae assessed was, *n* = 18 for AFW, *n* = 18 for BBJD, and *n* = 19 for SCW. All treatment groups were fed identically, with each container receiving 0.1 g of crushed TetraFin Goldfish Flake Food (Tetra Holding US, VA, USA) ∼48hr post transfer into AFW, BBJD, or SCW.

### 2.7 Statistics

All data sets were analyzed using Prism^ࣨ^ 9 software (GraphPad Software Inc., CA, USA). Each qPCR data set, including the *Craqp2* expression profile and effects of de-icers on *Craqp2* transcript in each organ, was analyzed with a one-way ANOVA with multiple comparisons following log-transformation. An unpaired *t*-test was used to determine significance between ds*βLac*-treated and ds*AQP2*-treated midges to determine significance of knockdown efficiency of *Craqp2* transcript. For survival and body water data, a one-way ANOVA with multiple comparisons was completed to determine significant differences between ds*βLac*-treated and ds*AQP2*-treated midge larvae.

## 3 Results

### 3.1 **Characterization of Cr**AQP**2**

**Fig. 1:**
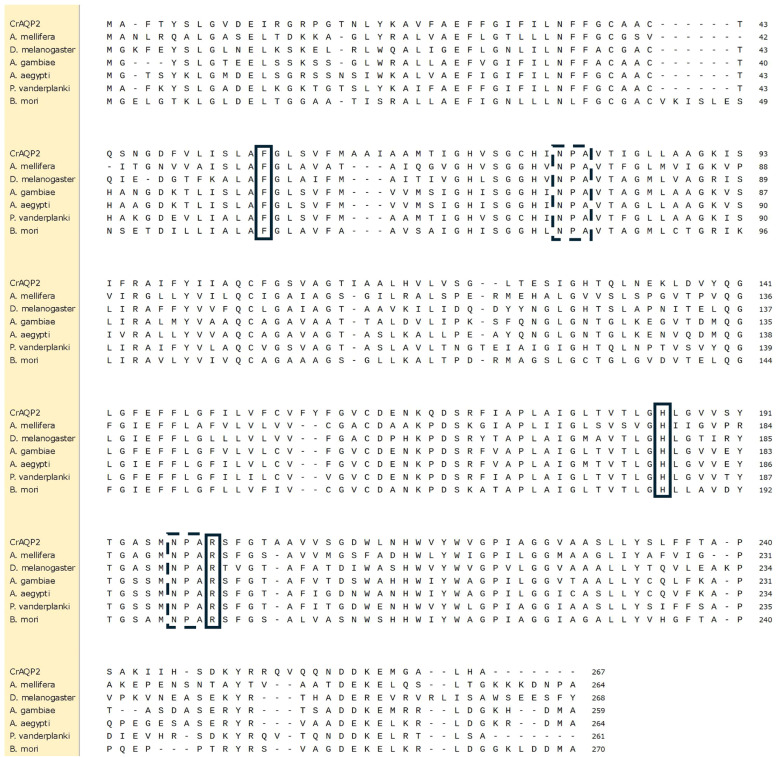
**Multiple sequence alignment of the *Chironomus riparius* aquaporin CrAQP2 (PRIP) deduced protein sequence with select orthologs from other insects.** CrAQP2 shares sequence similarity with characterized insect PRIPs including: *Apis mellifera* (XP_394,391.1), *Drosophila melanogaster* (NP_610,686.1), *Anopheles gambiae* (BAI60044.1), *Aedes aegypti* (XP_001656932.1), *Polypedilum vanderplanki* (BAF62090.1), *Bombyx mori* (NP_001153661.1). Dashed boxes identify the two NPA motifs and solid boxes identify the selectivity filter aromatic-arginine residues (ar/R).

The deduced amino acid sequence of CrAQP2 contains the hallmark features of aquaporins with the two NPA motifs that form the pore of the channel and the selective filter-forming aromatic-arginine residues (Fig. 1). The CrAQP2 sequence is similar to the sequences of characterized PRIP aquaporins as shown by sequence alignment with several PRIP aquaporin homologs from other insects.

### 3.2 *Craqp2* transcript abundance in osmoregulatory organs

**Fig. 2:**
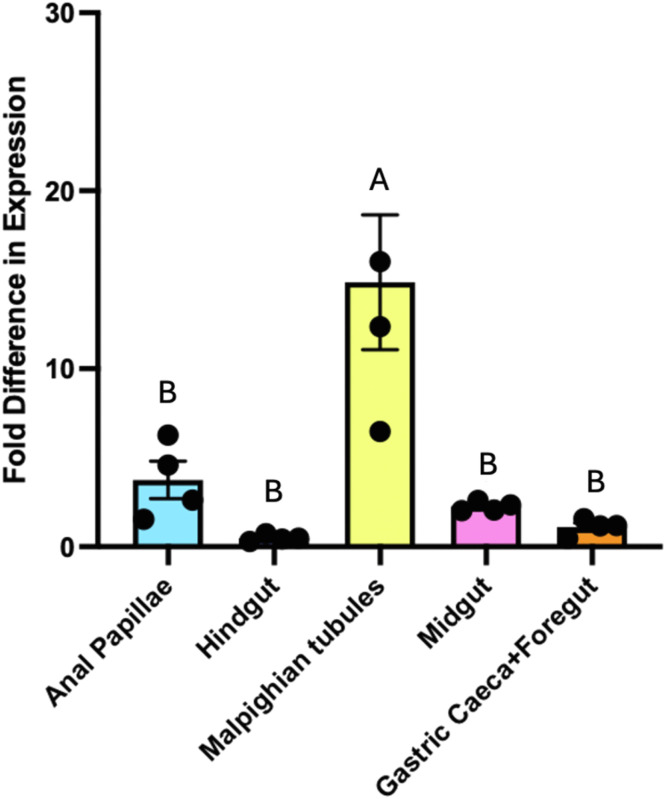
***CrAQP2* expression profile in the osmoregulatory organs of *Chironomus riparius* larvae in freshwater.** Larval freshwater *Chironomus riparius* aquaporin-2 (*CrAQP2*) transcript abundance in the osmoregulatory organs, including the gastric caeca with foregut (GC+FG), midgut (MG), Malpighian tubules (MTs), hindgut (HG), and anal papillae (AP). *CrAQP2* was detected in each of the organs studied, with highest transcript abundance in the MTs and lowest abundance seen in the HG tissue relative to the GC+FG as the calibrator. Bars show mean (± SEM) values with different letters denoting significantly different *CrAQP2* transcript abundances between organs as determined by one-way ANOVA with Tukey’s multiple comparison’s test (*p* < 0.05), *n* = 3–4.

*Craqp2* transcript was detectable in each of the osmoregulatory organs examined (Fig. 2). The MTs had the highest levels of *Craqp2* transcript, which was significantly higher than all other osmoregulatory organs analyzed with at least 5 times greater abundance. Comparatively, *Craqp2* transcript abundance in all other examined osmoregulatory organs showed no statistical difference, although the trend indicated moderate levels in the AP and MG, while HG and GC/FG had the lowest abundance.

### 3.3 **Immunolocalization of Cr**AQP**2 in larval *C. riparius***

**Fig. 3:**
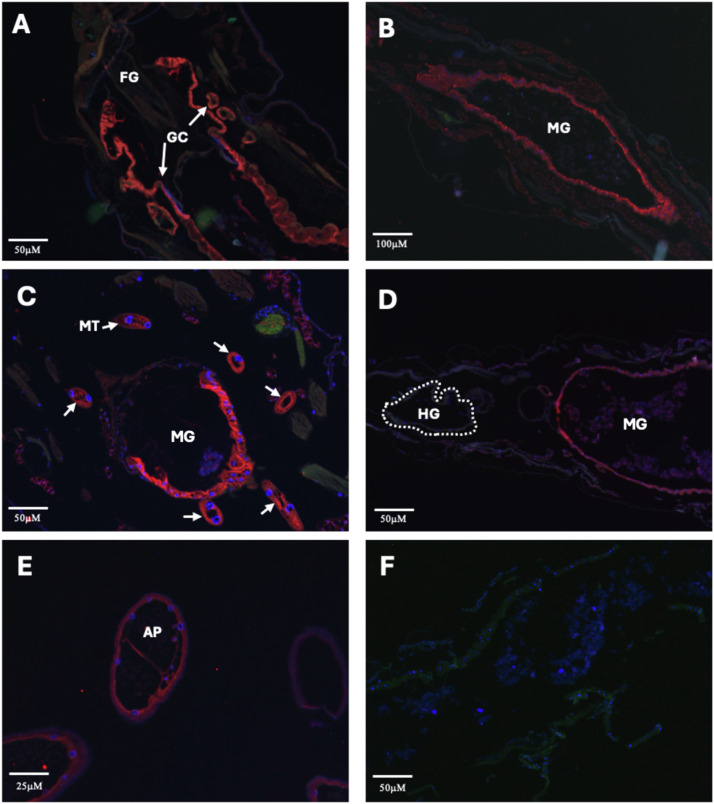
**Immunolocalization of AQP2 in the osmoregulatory organs of 4th instar larval *Chironomus riparius* midges reared in freshwater. A.** Gastric caeca (GC) and foregut (FG), **B.** midgut (MG), **C.** Malpighian tubules (MT), **D.** hindgut (HG), and **E.** anal papillae (AP). Panel **F.** shows the negative control with primary antibody omitted, where no AQP2 staining was observed over the whole-body midge section. For each organ, at least 3–4 biological replicates were completed each with 4–5 technical replicates run. Red staining indicates AQP2 immunoreactivity; green staining indicates the Na^+^/K^+^-ATPase (NKA) membrane marker immunoreactivity; and blue (DAPI) staining for nuclei.

We utilized a custom antibody raised against a peptide antigen representing a small portion of AaAQP2, the AQP2 homolog of *Aedes aegypti* (Durant et al., 2021). This antigen sequence is similar to the putative CrAQP2 sequence (see Supplementary Figure S1). Using this antibody, AaAQP2-like immunoreactivity was detected in the osmoregulatory organs, including the GC+FG, MG, MTs, and AP (Fig. 3). Although immunohistochemistry was completed for organs of larvae exposed to AFW, BBJD and SCW, no differences were observed in AaAQP2-like immunoreactive staining between treatments, therefore representative images from AFW larvae were chosen to summarize the localization of AaAQP2-like immunoreactivity in osmoregulatory organs (Fig. 3). Samples processed from BBJD and SCW-treated midges had similar relative AaAQP2-like immunoreactive staining (see Supplementary Figure S2). Beginning with the anterior portion of the alimentary canal, AaAQP2-like immunoreactivity was detected in the GC but not in the FG region, with staining appearing uniform within the basolateral membrane of the GC (Fig. 3A). AaAQP2-like immunolocalization was also visible in the MG tissue, with staining mostly appearing at the apical or lumen facing membrane of the gut cells (Fig. 3B). Transverse cross sections of the MTs show mainly cytosolic as well as apically localized AaAQP2-like immunoreactive staining (Fig. 3C). In the HG, there was no AaAQP2-like immunoreactivity detected (Fig. 3D). Finally, AaAQP2-like immunoreactivity was detected within the basal membrane of the externally located AP organ, with no staining visible at the apical membrane (Fig. 3E). The negative control, which excluded the primary antibody, had no AaAQP2-like immunoreactivity in any region of the whole-body midge (Fig. 3F).

### 3.4 effects of de-icers on *CrAQP2* transcript abundance in midge osmoregulatory organs

**Fig. 4:**
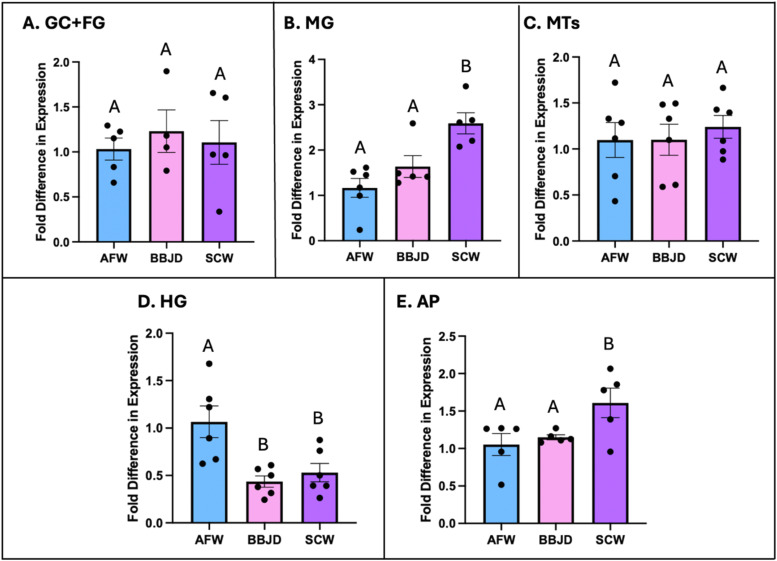
**Analysis of *CrAQP2* expression in the osmoregulatory organs of larval *Chironomus riparius* in response to road de-icers.** Transcript abundance of *CrAQP2* in the osmoregulatory organs of the midge following acute (24hr) exposures to artificial freshwater (AFW), brine beet juice de-icer (BBJD), and NaCl-contaminated water (SCW). **A.***CrAQP2* abundance in the gastric caeca with foregut (GC+FG) tissue, showing no changes in transcript abundance with de-icer treatment (*n* = 4–5). **B.***CrAQP2* abundance in the midgut (MG), which increased in SCW relative to the AFW and BBJD groups (*n* = 5–6). **C.***CrAQP2* abundance in the Malpighian tubules (MTs), showing no changes in transcript abundance with de-icer treatment (*n* = 6). **D.***CrAQP2* abundance in the hindgut (HG), which decreased in BBJD and SCW relative to the AFW group (*n* = 6). **E.***CrAQP2* abundance in the anal papillae (AP), which increased in SCW relative to the AFW and BBJD groups (*n* = 5). For each graph, values are displayed as mean±SEM and differences analyzed by one-way ANOVA with Tukey’s multiple comparisons where different letters denote significantly different transcript abundance (*p* < 0.05).

Acute exposure (24 hr) to BBJD or SCW did not alter the *CrAQP2* transcript abundance in the GC+FG and MTs (Fig. 4A& 4C). In the MG and AP, SCW treatment significantly increased the *CrAQP2* transcript abundance relative to AFW; however, BBJD did not affect *CrAQP2* transcript levels in these organs (Fig. 4B& 4E). In the HG, *CrAQP2* transcript abundance was significantly reduced with both BBJD and SCW treatment compared to AFW (Fig. 4D).

### 3.5 Influence of CrAQP2 knockdown on total body water content under exposure to de-icers

**Fig. 5:**
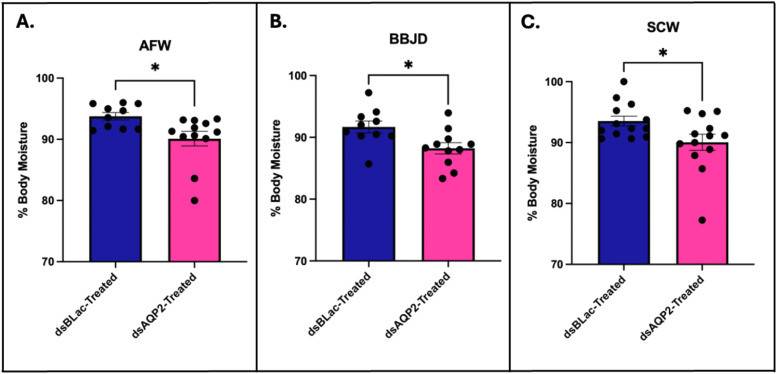
**Total body water post *CrAQP2* knockdown in larval *Chironomus riparius* post transfer to AFW, BBJD, and SCW**. Body water percentage ( %) was measured after *CrAQP2* knockdown and a subsequent acute (24hr) exposure to artificial freshwater (AFW), brine beet juice de-icer (BBJD), or NaCl-contaminated water (SCW). As a knockdown control, dsβLac-treatment was used for all conditions. **A**. Body water % post AFW transfer, showing significant decrease in body water with *CrAQP2* knockdown. **B**. Body water % post BBJD transfer, showing a significant decrease in body water with *CrAQP2* knockdown. **C**. Body water % post SCW transfer, showing significant decrease in body water with *CrAQP2* knockdown. For all panels (**A-C**), individual unpaired *t*-tests were completed and an * represents *p* < 0.05 (*n* = 10–13).

Total body water following *CrAQP2* knockdown in *C. riparius* larvae exposed to de-icers was measured. Body water percentage was determined following *CrAQP2* knockdown with acute (24hr) exposures to AFW, BBJD, or SCW. Larvae exposed to AFW with *CrAQP2* knockdown experienced a significant decrease in body water in comparison to the ds*ßLac*-treated individuals (Fig. 5A). Similarly, *CrAQP2* knockdown resulted in a significant decrease in body water in comparison to ds*ßLac*-treated controls following both BBJD (Fig. 5B) and SCW exposure (Fig. 5C).

### 3.6 Influence of *CrAQP2* knockdown on midge survival under prolonged exposure to de-icers

**Fig. 6:**
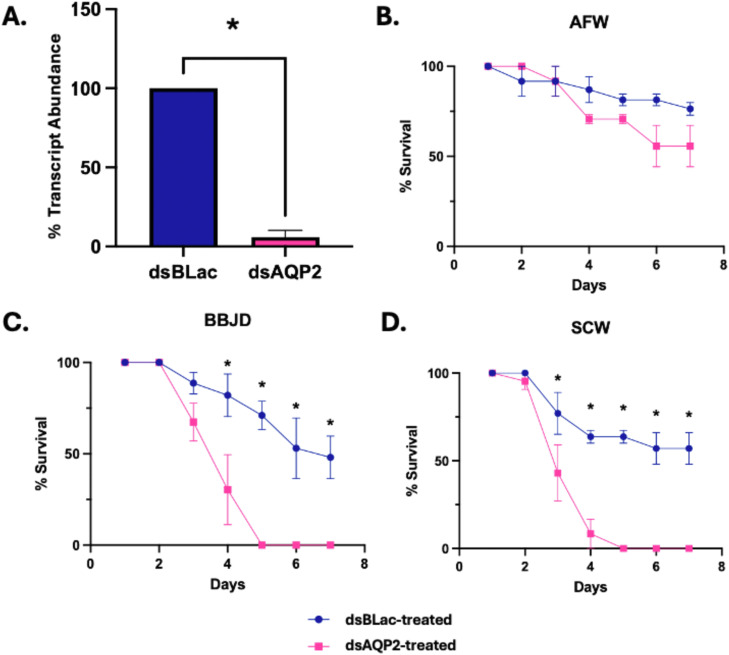
***Chironomus riparius* survival post *CrAQP2* knockdown and following prolonged exposure to road de-icers.** Midge larvae survival was observed over a 7-day period, following *CrAQP2* knockdown and exposure to artificial freshwater (AFW), brine beet juice de-icer (BBJD), or NaCl-contaminated water (SCW), with mean±SEM values reported. All treatments were completed on both ds*AQP2*-treated and ds*βLac*-treated larvae, with the latter serving as a knockdown control with larvae treated identically. **A.** Verification of *CrAQP2* gene knockdown in midge larvae, 48hr post dsRNA treatment. **B.** Survival ( %) of midge larvae in AFW for ds*βLac*-treated (*n* = 17) and ds*AQP2*-treated (*n* = 18) individuals. Survival between control and knockdown larvae was statistically similar over the 7-day period. **C.** Survival ( %) of midge larvae in BBJD for ds*βLac*-treated (*n* = 18) and ds*AQP2*-treated (*n* = 18) individuals. Survival in the *CrAQP2* knockdown group was significantly reduced compared to the control larvae by 4-days post transfer to BBJD. **D.** Survival ( %) of midge larvae in SCW for ds*βLac*-treated (*n* = 17) and ds*AQP2*-treated (*n* = 19) individuals. Survival in the *CrAQP2* knockdown group was significantly reduced compared to the control larvae by 3-days post transfer to SCW. A two-way ANOVA with multiple comparisons was run for each set of values and data points with an asterisk (*) denote statistically significant differences (*p* < 0.05) between the ds*βLac*-treated and ds*AQP2*-treated larvae at each time point.

The survival of *C. riparius* larvae was assessed in *CrAQP2* knockdown larvae under prolonged exposure to de-icers. Larvae exposed to *CrAQP2* dsRNA showed significantly reduced transcript abundance (∼90 %) after 48 hr exposure to dsRNA where levels were just 10 % of those in the *ßlac* dsRNA control group (Fig. 6A). Survivability was assessed over one week post *CrAQP2* knockdown. When maintained in AFW, midge survival decreased over the course of 7 days in both ds*ßLa*c-treated and ds*AQP2*-treated larvae, with no significant differences observed between the two groups (Fig. 6B). When midges were transferred from AFW into BBJD, survival of ds*AQP2*-treated larvae was stable for the first 2–3 days post transfer, but significantly decreased relative to the ds*ßLac*-treated larvae 4 days post transfer, before no survival was seen by day 5 (Fig. 6C). Similarly, in the SCW exposure group, ds*AQP2*-treated larvae had survival comparable to ds*ßLac*-treated larvae for the first 2 days; however, a rapid decline in survivability was seen at 3-days post transfer with ∼50 % surviving, followed by <10 % survival at 4-days post transfer and no survival by day 5 post transfer (Fig. 6D).

## 4 Discussion

### 4.1 Overview

Salinization of freshwater is a serious global issue and, in temperate climates, the use of de-icing agents during the winter season contributes to the problem. Thus, salinization of aquatic environments can have detrimental consequences to organisms living in freshwater, including aquatic insects, which are important in maintaining a healthy ecosystem. Although a number of studies have examined the effect of salinization on ion transport in freshwater insects (Kapoor et al., 1978; Jonusaite et al., 2011, 2013; Scheibener et al., 2016; Jonusaite et al., 2017; Nowghani et al., 2019; Orr and Buchwalter, 2020; Lambret et al., 2021; Picinic et al., 2022), comparatively little work has been done on water transport. In this study, we identified a putative water selective aquaporin (AQP) in *C. riparius* larvae which we named CrAQP2 and shares ∼70 % sequence identity with PRIP AQPs of other insects. We conclude that CrAQP2 is a homolog of the *Pyrocoelia rufa* integral protein (PRIP) (Cabrero et al., 2020; Drake et al., 2010; Yang and Piermarini, 2017; Tan and Chen, 2023). We then examined the effects of de-icing agents including NaCl and brine-beet juice de-icer (BBJD) on CrAQP2 expression and function.

We found CrAQP2 gene transcript and protein to be present in almost all of the osmoregulatory organs of midge larvae that were raised in their typical freshwater environment simulated in the laboratory by artificial freshwater (AFW). Studies on these PRIP channels have determined that these aquaporins transport water and some, but not all, can also transport urea or glycerol and therefore this suggests that the function of CrAQP2 is primarily for water transport (Azuma et al., 2012; Drake et al., 2015; Goto et al., 2011; Kikawada et al., 2008; Fabrick et al., 2014). This assumption is consistent with CrAQP2 having the highly conserved residues, phenylalanine, histidine, and arginine of the selectivity filter (see Fig. 1). Therefore, under this assumption that CrAQP2 transports water, we propose some functional roles for this transporter when *C. riparius* larvae are exposed to de-icing agents.

### 4.2 Expression of CrAQP2 in osmoregulatory organs of larvae in AFW, SCW and BBJD

#### 4.2.1 Malpighian tubules

The highest abundance of *CrAQP2* transcript was observed in the MTs of larval *C. riparius* and we found putative CrAQP2 (AaAQP2-like staining) protein dispersed throughout the cytosol and along the apical membrane of the MT cells (Fig. 2C). This observed staining pattern is consistent with CrAQP2 localization in the larger more abundant principal cells of MTs. In *D. melanogaster,* PRIP is highly expressed in the stellate cells of the MTs, aiding in transepithelial water entry into the lumen (Cabrero et al., 2020); whereas in adult *A. aegypti*, AaAQP2 (PRIP homolog) is expressed in both the principal and stellate cells of the MTs (Drake et al., 2010; Picinic et al., 2024). The MTs play an important role in both aquatic and terrestrial insects to produce primary urine, while maintaining haemolymph osmolarity. For *C. riparius* larvae that inhabit freshwater, the MTs must consistently produce dilute urine to clear excess water as a result of their hypoosmotic environment and CrAQP2 is positioned to participate in this process by transporting water into the lumen of the MTs. With de-icer treatment (BBJD and SCW), which decreases the gradient for water entry into the body but increases salt uptake, we found no changes to *CrAQP2* transcript abundance, nor did we find any qualitative differences in AaAQP2-like staining in the MTs. In *A. aegypti* larvae reared in brackish water (30 % seawater; NaCl the predominant salts), transcript abundance of aquaporins (including AaAQP2, the PRIP homolog) in MTs was no different than the levels measured in freshwater reared larvae (Misyura et al., 2020). Furthermore, the protein abundance of at least three distinct aquaporins in the MTs, excluding AaAQP2 which was not assessed, were consistent between freshwater and brackish water reared larvae (Misyura et al., 2020). Relatedly, fluid secretion rates of MTs from brackish water mosquito larvae were found to be comparable to those from freshwater larvae (Donini et al., 2006). Similarly, the MTs from *C. riparius* larvae reared in SCW (NaCl) had fluid secretion rates similar to those of AFW-reared larvae (Reinsborough and Donini, 2025). Interestingly, MTs from *C. riparius* larvae exposed to BBJD secreted fluid at rates 1.5 times those of MTs from AFW and SCW exposed larvae and there is some evidence that this aids in removing the excess K^+^ which the larvae encounter in the BBJD (Reinsborough and Donini, 2025). Collectively, the results from the mosquito and midge larvae suggest that the underlying expression of AQPs is sufficient to support different rates of fluid secretion; that other AQPs that have yet to be discovered may play a role in the increased fluid secretion rates observed in BBJD midge larvae; or that AQPs (including CrAQP2) may be regulated through post translational modifications. In terms of the latter, the phosphorylation state of AQPs in adult female mosquito MTs did not change after a blood meal despite the dramatic increase in fluid secretion that ensues (Kandel et al., 2022).

#### 4.2.2 Anal papillae (AP)

*CrAQP2* transcript as well as AaAQP2-like immunoreactive staining in the AP of midge larvae were detected. The AP are important osmoregulatory organs in mosquito and midge larvae, responsible for the active uptake of ions and osmotically obliged water from the external freshwater environment (Jarial, 1995; Nguyen and Donini, 2010; Marusalin et al., 2012). Transcript levels of several AQPs were measured in the AP of *A. aegypti* and AaAQP2 transcript abundance was high compared to other AQPs when larvae were held in freshwater (Marusalin et al., 2012; Akhter et al., 2017; Durant et al., 2021). Since AaAQP2 is the predominantly expressed water transporting AQP in the AP it is likely to be, at the very least, partially responsible for the observed water flux across the AP epithelium (Marusalin et al., 2012; Akhter et al., 2017; Durant et al., 2021). Therefore, CrAQP2 is also likely to be important in mediating water transport across the AP epithelium of *C. riparius*, since we found AaAQP2-like immunolocalization on the basal membrane of the midge AP (Fig. 2E).

With respect to the influence of de-icers, it is already known that when mosquito larvae are exposed to salinity, the AP undergo morphological and ultrastructural changes (Wigglesworth, 1938; Sohal and Copeland, 1966; Surendran et al., 2018). It appears that when larvae are exposed to NaCl, their AP decrease in size and the epithelium has fewer microvilli and membrane folds thereby reducing the surface area for transport (Wigglesworth 1938; Sohal and Copeland, 1966). However, when larvae are raised over at least 20 generations in diluted natural seawater the AP increase in size over subsequent generations (Surendran et al., 2018). Furthermore, the anal papillae can adjust their ion transport functions in response to salinity (Nguyen and Donini, 2010; Donini et al., 2007; Reinsborough and Donini, 2025). In *A. aegypti*, exposure to NaCl based salinity decreases Na^+^ uptake by AP and in *C. riparius* this leads to an apparent reversal of transport from Na^+^ uptake to Na^+^ secretion (Donini et al., 2007; Reinsborough and Donini, 2025). When *C. riparius* were exposed to BBJD which contains relatively less NaCl, the AP responded similarly to those in the mosquito by decreasing, but not reversing, Na^+^ uptake (Reinsborough and Donini, 2025).

In this study, SCW caused a significant increase in *CrAQP2* transcript abundance in midge AP while BBJD exposure did not change *CrAQP2* transcript levels. The effects of increased NaCl-based salinity such as brackish water exposure caused similar findings in *A. aegypti* larvae, where a significant increase in AaAQP2 protein was determined in mosquito AP, relative to the freshwater control (Durant et al., 2021). The haemolymph of *C. riparius* remains hyperosmotic to the surrounding SCW (haemolymph: ∼ 270 mOsmol versus SCW: ∼ 240 mOsmol; Reinsborough and Donini, 2025); however, the gradient at which water entry is favoured is decreased with the increased external osmolarity from a difference of ∼ 160 mOsmol in AFW to ∼30 mOsmol in SCW (Reinsborough and Donini, 2025). In BBJD, the difference between haemolymph (hyperosmotic to BBJD) and the BBJD is ∼ 80 mOsmol (Reinsborough and Donini, 2025). Therefore, it stands to reason that a greater influx of water across the AP is occurring in BBJD relative to SCW conditions, and the level of water influx in BBJD may be sufficient to deal with the increased fluid secretion by the MTs in BBJD (Reinsborough and Donini, 2025). In contrast, the lower influx of water at the AP that would be caused by the reduced osmotic gradient in SCW may not be sufficient to avoid net water loss with the MTs maintaining rates of fluid secretion similar with those in AFW. This may be the reason why larvae in SCW have increased expression of *CrAQP2* in the AP, so as to increase the influx of water when faced with a lower osmotic gradient in an attempt to avoid dehydration.

#### 4.2.3 Midgut, gastric caeca, and hindgut

*CrAQP2* transcript and AaAQP2-like immunoreactivity were evident in the MG and GC. Given that the foregut mainly serves as a conduit for food passage to the MG, and the absence of AaAQP2-like immunoreactivity in sections of the foregut, we suggest that the *CrAQP2* expression in the GC-FG samples is confined to the GC. In the hindgut, *CrAQP2* transcript was detectable but at relatively low levels and AaAQP2-like immunoreactivity was not observed.

In *A. aegypti*, AaAQP2 transcript was detected in the midgut of larvae and adults, and AaAQP2 was immunolocalized to the basolateral epithelial membrane of the posterior midgut in adults (Drake et al., 2010; Marusalin et al., 2012; Picinic et al., 2024). The tissue sections probed in this study showed AaAQP2-like immunoreactivity mostly confined to the apical (lumen facing) side of the *C. riparius* MG. We believe that most of the sections probed were from the anterior regions of the MG and cannot draw any conclusions about the localization of AaAQP2-like staining in the posterior MG. In some adult mosquitoes such as *A. aegypti* (Drake et al., 2010; 2015) and *Anopheles sinensis* (Zhao et al., 2024), entomoglyceroporins that transport solutes preferentially over water are more highly expressed in the MG presumably aiding in absorption of solutes into the haemolymph. At this stage, information on the identity or expression of other AQPs in *C. riparius* midgut remains unknown, although it is likely that entomoglyceroporins are present and expressed in the midge. The midgut of insects has a peritrophic membrane which isolates the food bolus from direct contact with the epithelial cells. As the food progresses posteriorly, enzymes are secreted, and digestion occurs. Fluid then travels anteriorly within the space between the peritrophic membrane and the epithelium where absorption of nutrients can occur (Ramsay, 1950; Volkmann and Peters, 1989). In *C. riparius*, CrAQP2 in the MG may contribute to sustaining this flow of fluid, supporting digestive and absorptive processes and/or mediating absorption of water into the haemolymph.

We also found increases in *CrAQP2* transcript abundance in the MG with SCW treatment relative to AFW; however, no changes were seen with BBJD treatment. As larvae imbibe their external medium when feeding, the resulting osmotic gradient across the midgut would be greatly reduced in SCW resulting in less water absorption into the haemolymph (Reinsborough and Donini, 2025). Larvae face an accumulation of NaCl in SCW. If CrAQP2 is involved in water uptake across the MG in the midge, it is possible that in response to SCW exposure, the larval midges upregulate *CrAQP2* levels in the MG to increase the uptake of water into the haemolymph helping to avoid dehydration caused by excess ion uptake and the reduction in the osmotic gradient that drives water towards the haemolymph. In BBJD, the osmotic gradient is not reduced to the same levels as in SCW and therefore it may be unnecessary to increase *CrAQP2* expression to maintain sufficient water uptake into the haemolymph across the MG (Reinsborough and Donini, 2025).

Some of the fluid that moves anteriorly between the peritrophic membrane and epithelium of the midgut enters the gastric caeca, sites that are implicated in the final digestion of proteins and carbohydrates as well as ion and solute exchange with the haemolymph (see Godoy et al., 2023). In the GC of *C. riparius*, CrAQP2 localized on the basolateral membrane of epithelial cells can regulate water exchange with the haemolymph perhaps facilitating digestion and absorption of nutrients. In larval mosquitoes reared in freshwater, the GC move Na^+^ and K^+^ from the haemolymph into the GC lumen (D’Silva et al., 2017). Along with active ion transporters, AaAQP2 transcript (and other AQPs) are expressed in the GC of freshwater larval *A. aegypti* (Patrick et al., 2006; Misyura et al., 2020). In light of these facts, it is possible that water from the haemolymph is osmotically obligated to follow ion transport, similar to what occurs at the MTs. If this is the case, the GC may help to rid the larvae of excess water when they are in a freshwater environment. Assuming the GC of *C. riparius* function similarly to those in the mosquito, CrAQP2 could facilitate this movement of water. However, at this time, we do not know what the osmolality of the GC luminal contents are, and this information is necessary to draw a conclusion on this point.

The BBJD and SCW treatments did not affect the abundance of *CrAQP2* transcript in the larval midge GC. It is plausible that the GC consistently secretes ions and water into their lumen irrespective of changes in external salinity. In *A. aegypti* larvae reared in brackish water, there is a reduction in ATPase activity and overall preference for secreting Na^+^ over K^+^ across the caecal epithelium (D’Silva et al., 2017). However, it was demonstrated that the transepithelial potential, as well as the sum of cation (Na^+^ and K^+^) concentrations, did not change in the caeca of brackish water larvae. Furthermore, AaAQP2 mRNA abundance in larval *A. aegypti* GC did not change following exposure to brackish water (Misyura et al., 2020). This suggests that similar rates of water secretion occur; however, since the mosquito larvae ingest the water they live in, the osmotic gradient across the GC of brackish water larvae may be significantly lower than freshwater larvae if that brackish water enters the GC lumen without much modification (D’Silva et al., 2017). A similar scenario is likely occurring in the GC of *C. riparius* larvae exposed to SCW or BBJD, where *CrAQP2* expression remains consistent relative to freshwater exposed larvae.

In this study, we did not detect high abundance of *CrAQP2* in the HG, which is consistent with previous studies on *A. aegypti* larvae that reported extremely low levels of AaAQP2 in the HG, relative to the other AQP isoforms expressed in this organ (Marusalin et al., 2012). Due to their freshwater environment, the HG is likely working to actively re-uptake ions while leaving water in the final urine destined for excretion (Bradley and Phillips, 1977; Jonusaite et al., 2013); therefore, it suggests that CrAQP2 may not play a prominent role in the HG of *C. riparius* larvae. Despite this low baseline transcript abundance and absence of detectable immunoreactivity in AFW, we measured the effects of BBJD and SCW on *CrAQP2* expression in the HG of *C. riparius*. De-icer treatment significantly decreased the already very low levels of *CrAQP2* in the HG, relative to AFW and immunohistochemistry revealed little to no presence of CrAQP2 in the HG. Therefore, water flux across the HG epithelium is likely to be low so that excess water remains in the urine for excretion. However, further investigation into potentially other AQPs present is essential to understanding the role of the HG.

### 4.3 Role of CrAQP2 in total body water content with de-icer treatment

To understand the role of CrAQP2 in the response of *C. riparius* larvae to de-icer treatment on a whole organism level, we decreased the expression of *CrAQP2* in AFW larvae using RNA interference. We then subjected these larvae to an abrupt, acute exposure SCW or BBJD, in comparison to control larvae maintained AFW, in an attempt to mimic what they may encounter in their natural environment as a result of salinization. After the exposure, total body water was measured to assess the contribution of CrAQP2 to the osmotic regulation of larvae. Decreasing the expression of *CrAQP2* significantly decreased the total body water of larvae, regardless of treatment (including controls).

In AFW larvae, we reason that the AP are predominantly responsible for the reduction in body water following *CrAQP2* knockdown because the AP are sites of water uptake in dilute conditions and moderately express *CrAQP2* transcript (Fig. 2; Marusalin et al., 2012). It is likely that other AQPs are expressed in the AP, however; if the AP of *C. riparius* are similar to the AP of *A. aegypti* then expression of other AQPs is low (Durant et al., 2021). Therefore, decreased *CrAQP2* expression in the AP is expected to significantly reduce water uptake. The MG may also contribute to reduced water uptake when *CrAQP2* expression is compromised assuming this transporter mediates water absorption across the MG (see explanations above, Section 4.2.3). On the other hand, any reduction in *CrAQP2* expression in the MTs or GCs would likely cause the accumulation of water in the body since both of these organs are normally responsible for secreting water. However, it is possible that the MTs continue to secrete primary urine at a consistent rate even with *CrAQP2* knockdown because of the likelihood of other AQPs expressed by the MTs. For example, in the mosquito *A. aegypti*, four of the six AQP genes have been localized in the MTs (Drake et al., 2010; Marusalin et al., 2012; Misyura et al., 2020; Picinic et al., 2024). This suggests a model where water transport may occur through many AQPs simultaneously and therefore the reduction in *CrAQP2* expression alone may not have a significant effect on the overall function of the MTs if this were the case. As a result, this suggests that the lower body water observed with *CrAQP2* knockdown could be driven by the decreased capacity for water uptake at the AP.

Larvae exposed to de-icers face an osmotic gradient across the body wall which favours water movement into the haemolymph; however, this gradient is much lower than what they face in freshwater, particularly in SCW (see Section 4.2.2). Therefore, less water is obliged to enter through the AP and/or MG and the larval osmoregulatory physiology responds by increasing transcript abundance of *CrAQP2* in AP and MG in SCW exposure (Fig. 4) to increase water uptake (see Sections 4.2.2 & 4.2.3). *CrAQP2* knockdown under these conditions attenuates the physiological response resulting in insufficient water being taken up across AP and/or MG, and since the MTs likely continue to produce primary urine at similar levels, the larvae experience dehydration leading to significantly lower total body water content. Indeed, this is supported by recent evidence demonstrating that the MTs of larvae in SCW and BBJD secrete fluid at the same and higher rates than those in AFW, respectively (Reinsborough and Donini, 2025), and the effect of *CrAQP2* knockdown in MTs is likely to be blunted by the expression of other AQPs. Thus, the net result is similar to control larvae where dehydration occurs.

### 4.4 Effect of *CrAQP2* knockdown on larval survival

With the knowledge that *CrAQP2* knockdown causes dehydration in the midge larvae after just 24 h of exposure to de-icers and control conditions, we tracked survival of midges over 7 days. After 3 days, knockdown larvae in AFW showed lower survival than control larvae but at least half of the larvae survived to 7 days. We suggest that, although uptake of water across the AP would be compromised, as evidenced by lower body water, the osmotic gradient is sufficient to drive enough water into the body to permit survival. In contrast, in SCW where the osmotic gradient is reduced, the data suggests that not enough water enters the body and this manifests in an abrupt drop in survival beginning on day 3. In BBJD, the osmotic gradient may be sufficient to sustain enough water uptake, but this is likely countered by the increase in fluid secretion rates by the MTs causing a more gradual drop in survival beginning on day 3. In both de-icers, all larvae succumb to the salinization treatment by day 5.

In conclusion, this study identified the first AQP in *C. riparius,* which we named CrAQP2, and also measured its transcript abundance and localized its distribution at the protein level using immunohistochemistry in the alimentary canal of midge larvae. Furthermore, the influence of common road de-icers (BBJD and NaCl) on *CrAQP2* expression in each of the osmoregulatory organs was observed and knockdown of *CrAQP2* revealed the role of this water channel in mitigating osmotic stress associated with salinization. We found that CrAQP2 is expressed broadly in the midge alimentary canal and overall, NaCl-contaminated water caused differential gene expression in a few of the osmoregulatory organs while BBJD only affected *CrAQP2* expression in the HG. However, with *CrAQP2* knockdown, treatment of midge larvae with either BBJD or NaCl was lethal after only a few days. A reduction in CrAQP2 functioning associated with knockdown results in reduced body water for midge larvae in freshwater, SCW, and BBJD. From this study, it is evident that CrAQP2 is an important water channel protein in *C. riparius*, with a role in homeostatic water transport. Furthermore, salinization of freshwater influences CrAQP2 expression and its role in osmoregulation. CrAQP2 appears to be vital in the ability of *C. riparius* to adapt to and survive in increased salinity caused by road de-icer contamination.

## Funding

This study was supported by Natural Sciences and Engineering Research Council of Canada Discovery Grants # RGPIN-2024-06188 to AD; and RGPIN-2020–06130 to JPP, Ontario Ministry of Colleges and Universities, Ontario Graduate Scholarship to BNP.

## Credit author statement

B. Picinic: designed experiments, carried out experiments, analyzed data, wrote the manuscript. A. Reinsborough, carried out experiments. S. Jonusaite, carried out experiments. J-P. Paluzzi: designed experiments, edited the manuscript. A. Donini: designed experiments, edited the manuscript, obtained funding

## Declaration of competing interest

The authors declare that they have no known competing financial interests or personal relationships that could have appeared to influence the work reported in this paper.

## Appendix Supplementary materials

Image, application 1

Image, application 2
