# Supplementary material for: Discovery of an aquaporin (CrAQP2) in the freshwater larval midge, Chironomus riparius and its role in response to road de-icers
Source: Curr Res Insect Sci. 2026 Jan 16;9:100123. doi: 10.1016/j.cris.2026.100123 (PMC12861059; doi:10.1016/j.cris.2026.100123)
Supplement: Supplementary file 2 [file mmc2.docx]

**Figure S1. *Aedes aegypti* AaAQP2 epitope alignment with the putative CrAQP2 epitope in *Chironomus riparius*.**

The custom AaAQP2 antibody antigen from *Ae. aegypti* is shown in an alignment with the deduced CrAQP2 epitope of *C. riparius*. The alignment was done using Geneious Software (Dotmatics, MA, USA) demonstrating ~50% similarity over the antigen spanning residues.


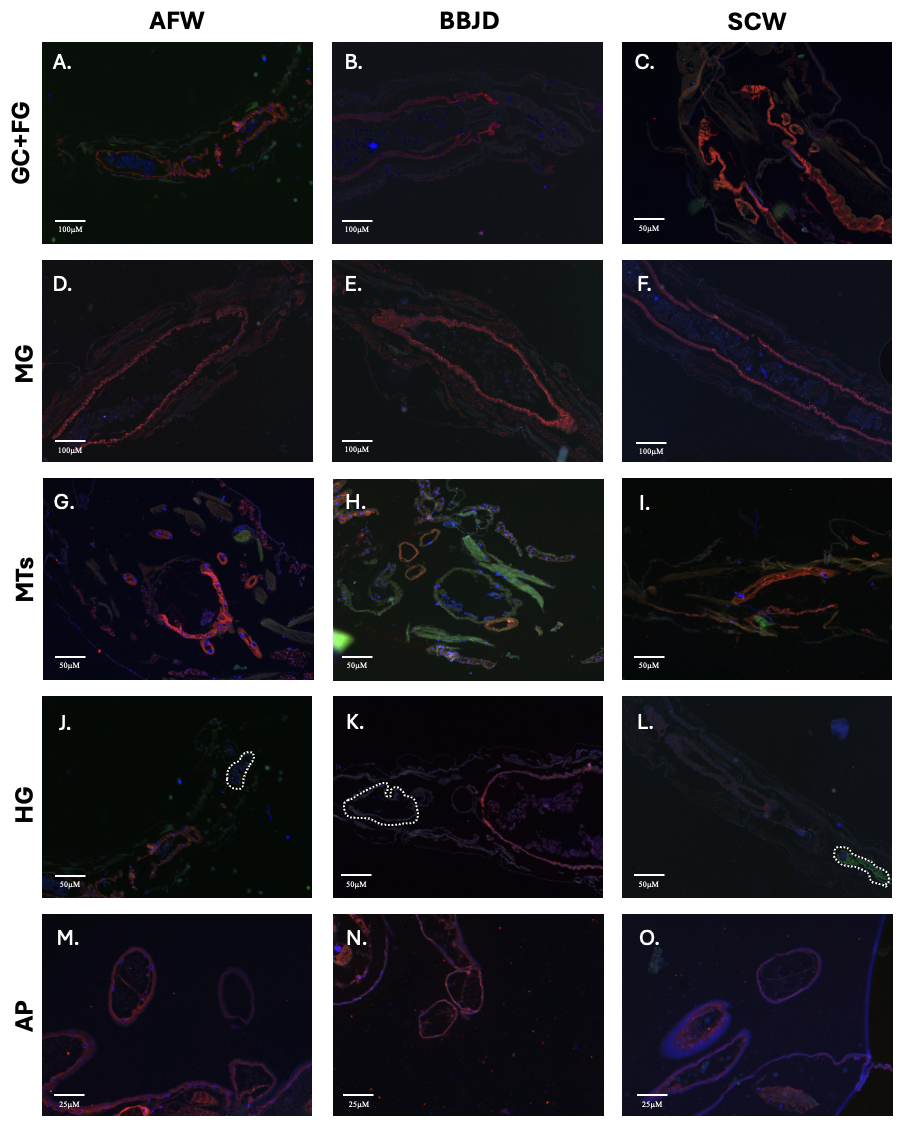


**Figure S2. Immunolocalization of CrAQP2 in osmoregulatory organs of larval *Chironomus riparius* in AFW, BBJD, and SCW.**

Localization of AQP2 in tissue sections of the osmoregulatory organs of 4^th^ Instar larval *C. riparius* midges in AFW, BBJD, and SCW including **A-C.** gastric caeca (GC) with foregut (FG), **D-F.** midgut (MG), **G-I.** Malpighian tubules (MTs), **J-L.** hindgut (HG), and **M-O.** anal papillae (AP). n=3-4 biological replicates for each organ, in addition to 4-5 technical replicates each. Red staining indicates AQP2 immunoreactivity; green staining indicates the Na^+^/K^+^-ATPase (NKA) membrane marker immunoreactivity; blue staining indicates the presence of individual nuclei.
